# Effect of a Peripheral Neuromodulation Protocol Combined with the Application of Therapeutic Exercise in Patients Diagnosed with Urinary Incontinence—A Study Protocol for a Randomized Controlled Trial

**DOI:** 10.3390/healthcare13141759

**Published:** 2025-07-21

**Authors:** Jesica Leal-García, Paula Blanco-Giménez, Eloy Jaenada-Carrillero, Marta Martínez-Soler, Borja Huertas-Ramírez, Alex Mahiques-Sanchis, Juan Vicente-Mampel

**Affiliations:** Department of Physiotherapy, School of Medicine and Health Sciences, Catholic University of Valencia, 46001 Torrent, Valencia, Spain; jesica.leal@ucv.com (J.L.-G.); eloy.jaenada@ucv.es (E.J.-C.); marta.martinez@ucv.es (M.M.-S.); borja.huertas@mail.ucv.es (B.H.-R.); alex.mahiques@mail.ucv.es (A.M.-S.); juan.vicente@ucv.es (J.V.-M.)

**Keywords:** overactive bladder, urinary incontinence, transcutaneous electric nerve stimulation, exercise

## Abstract

**Introduction:** Overactive bladder (OAB) and urinary incontinence (UI) are prevalent, particularly in older adults, and affect quality of life. OAB involves urgency, frequency, nocturia, and urgency incontinence, often linked to involuntary detrusor contractions. Treatment guidelines recommend a stepwise approach, starting with pelvic floor muscle training (PFMT), followed by pharmacological or minimally invasive therapies, such as neuromodulation. However, the combined effects of PFMT and neuromodulation have not been well established. This study aimed to evaluate the impact of combining pelvic floor exercises with neuromodulation versus PFMT with sham neuromodulation or standard physiotherapy after a 12-week intervention in individuals with OAB and UI. **Methods/Materials:** A double-blind, randomized controlled trial was designed with three groups: PFMT + neuromodulation, PFMT + sham, and conventional physiotherapy (control) in a 1:1:1 ratio. This study followed the CONSORT guidelines and was registered at ClinicalTrials.gov (NCT06783374). The sample size was calculated using GPower^®^ software, assuming a Cohen’s effect size of 1.04, a power of 0.80, an alpha of 0.05, and a 15% dropout rate, totaling 63 participants (21 per group). Participants attended 24 sessions over 12 weeks (2 sessions per week). The interventions were based on previously validated protocols. **Outcomes:** The primary outcomes included health-related quality of life, pelvic floor muscle function, pain, adherence, and general health. The secondary outcomes included Incontinence Quality of Life questionnaire, 3-day bladder diary, International Consultation on Incontinence Questionnaire–Urinary Incontinence Short Form, kinesiophobia, and electromyographic data.

## 1. Introduction

The International Continence Society (ICS) has established the standardized definition of overactive bladder (OAB) syndrome as the presence of urinary urgency, usually accompanied by frequency and nocturia, with or without urgency urinary incontinence, in the absence of urinary tract infections or other obvious pathology. The symptoms of an overactive bladder include four elements: urgency, frequent urination, nighttime urination, and urgent incontinence [1]. Epidemiological data from European countries indicate that the prevalence of overactive bladder (OAB) is higher in women than in men, with 21.9% of women affected. Additionally, OAB is more commonly observed in individuals who are overweight, obese, or aged ≥ 60 years [2]. The symptoms of OAB are caused by involuntary contractions of the detrusor muscle of the bladder during the filling phase of the micturition cycle [3], so urge urinary incontinence (UUI) is widely recognized as a well-known manifestation of OAB [1]. Although OAB is not always linked to detrusor overactivity (DO), as observed in urodynamic studies, it may present either as idiopathic (non-neurological) or as a result of a neurological condition, such as multiple sclerosis or spinal cord injury [4]. OAB can be categorized into two subtypes: “OAB-wet”, which is characterized by urinary incontinence, and “OAB-dry”, which occurs without incontinence [5,6].

The American Urological Association (AUA) clinical guidelines recommend a stepwise approach to the management of overactive bladder (OAB), progressing from less invasive to more invasive treatments based on the patient response [7]. Four primary lines of treatment have been established: noninvasive, pharmacotherapy, minimally invasive, and invasive therapies [8]. Noninvasive therapies primarily focus on incontinence management strategies. Among these, bladder training is strongly recommended for all patients owing to its proven efficacy, as supported by high-quality evidence from systematic reviews, including a Cochrane Review (2023) [9]. Specifically, intentional and focused contraction of the pelvic floor muscles (PFMs) during episodes of urgency, as demonstrated by systematic review findings [10], is maintained until the urgency subsides. Furthermore, multi-joint exercises that incorporate trunk stabilization with abdominal activation have been shown to effectively complement these interventions [11,12]. However, because these studies do not provide the systematization and planning of such interventions, a multidimensional strength training program that includes PFM contraction is needed [13]. Recently, anticipatory postural adjustments of upper and lower limb movements related to PFM have been documented, generated synergistically during involuntary and functional movements [14]. This approach enhances the muscular properties of the striated PFM through reflex responses that promote sensorimotor control of the posture, body awareness, and balance [15].

Due to the promising results, the effect of exercise combined with therapies is highly recommended for patients with persistent symptoms, as it can enhance symptom management and facilitate the appropriate adjustment of treatment based on individual outcomes [16]. Neuromodulation minimally invasive therapies represent effective treatment options for OAB in patients who exhibit inadequate responses to prior interventions or choose to discontinue these approaches. This is supported by a recent network meta-analysis comparing third-line therapies, including botulinum toxin injections, sacral neuromodulation, and percutaneous tibial nerve stimulation (PTNS), which identified these as the most representative and effective techniques [17]. Recently, there has been increased emphasis on the importance of shared decision-making when choosing the most suitable treatment or combination of treatments customized to meet the individual needs of the patient [8]. The combination of different techniques can provide a more comprehensive approach to symptom management in patients with OAB [18]. Previous studies have shown that combining pelvic floor muscle training (PFMT), transcutaneous tibial nerve stimulation, transvaginal electrical stimulation, and motor control exercises offers excellent safety and efficacy, provided that patients maintain long-term adherence to the treatment [8].

Neuromodulatory treatments for OAB and UI are applied at the suprapubic, transvaginal, sacral, and posterior tibial nerve levels [19]. Posterior tibial nerve neuromodulation (PTNN) is a more conservative approach with fewer complications, endorsed by the ICS with a Grade A recommendation internationally [1]. This technique can be performed in two ways: through percutaneous and transcutaneous electrical stimulation of the posterior tibial nerve [20]. It involves retrograde stimulation of pelvic nerves connected to the spinal cord via the sacral plexus in the S2 and S3 segments, where the motor branches of the sacral plexus (parasympathetic fibers) terminate in ganglion cells located in the bladder wall and detrusor muscle [20]. Data indicate that PTNS could lead to significant clinical improvement in patients with OAB with non-obstructive urinary retention [21]. Additionally, it has been observed that neuromodulation can be effective in treating urinary frequency, urgency, and nocturia, and in improving quality of life by around 30% [19]. OAB and UI are highly prevalent conditions that significantly affect quality of life. The efficacy of PFMT and neuromodulation in symptom improvement and urinary continence control is well established. However, the lack of standardized protocols and the need for more comprehensive therapeutic strategies justify further studies to optimize the clinical management of these conditions. Although Firinci et al. [16] conducted a randomized controlled trial supporting the combination of peripheral neuromodulation with PFMT, more protocols seek to improve this by incorporating a longer intervention period and standardized outcome measures, thereby enhancing the methodological rigor and clinical applicability of the findings. Hence, we hypothesized that pelvic floor strength training through a protocol, in combination with prior peripheral neuromodulation, may enhance the ability to inhibit detrusor contractions by utilizing involuntary perineal muscle contractions. The objective of this investigation was to assess the efficacy of combining pelvic floor muscle training with neuromodulation compared to pelvic floor muscle training with sham neuromodulation or conventional physical therapy after a 12-week treatment period in patients diagnosed with OAB and UI. The researchers hypothesized that pelvic floor strengthening, when administered as an adjunct to peripheral neuromodulation, would inhibit detrusor contractions by promoting involuntary contractions of the perineal musculature.

## 2. Materials and Methods

### 2.1. Study Design

**Figure 1:**
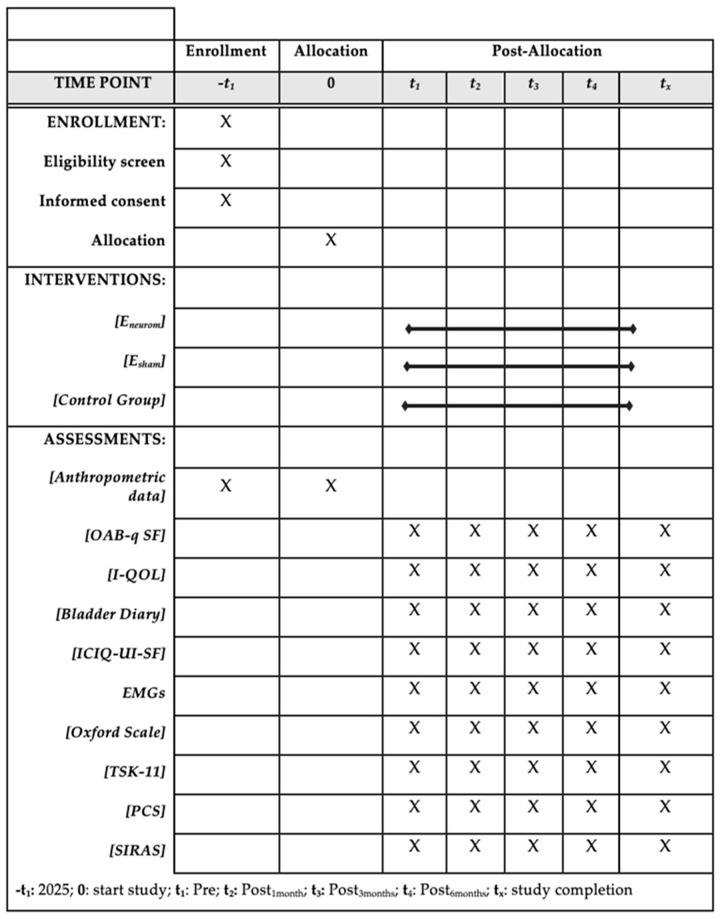
Execution schedule—recruitment, intervention, and reassessment.

This study adopts a prospective, double-blind, randomized controlled trial (RCT) design with a comparative longitudinal approach. The study protocol has been developed following the SPIRIT guidelines, which delineate the essential components that must be incorporated into randomized clinical trial protocols (Figure 1) [22]. The results will be communicated in accordance with the Template for Intervention Description and Replication (TIDieR) checklist to provide a detailed and accurate description of the intervention and its implementation [23].

This double-blind study will include three groups: (i) an exercise program combined with neuromodulation, (ii) an exercise program with a sham neuromodulation procedure, and (iii) a control group receiving conventional physiotherapy treatment, with participants equally distributed among the groups. The Ethics Committee of the Catholic University of Valencia approved the study protocol (13 January 2025) (ID: UCV/-2025/020), and the trial was registered in the ClinicalTrials.gov database under registration number NCT06783374 (20 January 2025). The timetable for completion of the study is as follows: the study will begin on 4 March 2025, with primary completion expected by 10 November 2026. The intervention will be conducted at the Clinics of the Catholic University of Valencia. Prior to the intervention, participants will receive a detailed video briefing that will explain the study’s objectives, procedures, and possible risks. All the participants who agree to participate will provide written informed consent forms prepared in accordance with the ethical principles outlined in the Declaration of Helsinki [24].

### 2.2. Study Population

The study will involve patients diagnosed with UUI and/or OAB who meet specific eligibility criteria, satisfy the clinical standards for PTNN, and are capable of adhering to an exercise regimen. While only one group will receive PTNN, all the participants, irrespective of their assigned groups (exercise + neuromodulation (E_neurom_), exercise + sham (E_sham_), and control group (conventional physiotherapy), must satisfy the same eligibility criteria. Recruitment efforts will include promoting “a new study on urinary incontinence” through flyers and posters distributed throughout Valencia, Spain, as well as through posts on widely used social media platforms, such as LinkedIn, Facebook, and Instagram. Moreover, we plan to implement community outreach, in-person information sessions, and collaboration with local health centers to ensure broader inclusion and improve access for populations. In fact, specialists in gynecology and nephrology play a key role in referring patients who have been diagnosed.

### 2.3. Inclusion and Exclusion Criteria

The criteria for inclusion are established as follows: (i) a diagnosis of urinary incontinence or an overactive bladder, (ii) women aged over 45 years, (iii) a condition persisting for at least three months, (iv) meeting the criteria for selection as a candidate for neuromodulation treatment (urge urinary incontinence), and (v) fluency in either Spanish or English. Participants will be excluded if they fulfill any of the following criteria: (i) any past or planned surgeries in the lumbar or abdominal regions, (ii) the presence of fractures or serious medical conditions, (iii) being pregnant or having the potential to become pregnant during the study period, (iv) any neurological or psychiatric disorders, (v) having stress urinary incontinence, (vi) women with autoimmune diseases or cancer, (vii) fear of needles, and (viii) individuals who have previously undergone neuromodulation treatment.

### 2.4. Procedure

**Figure 2:**
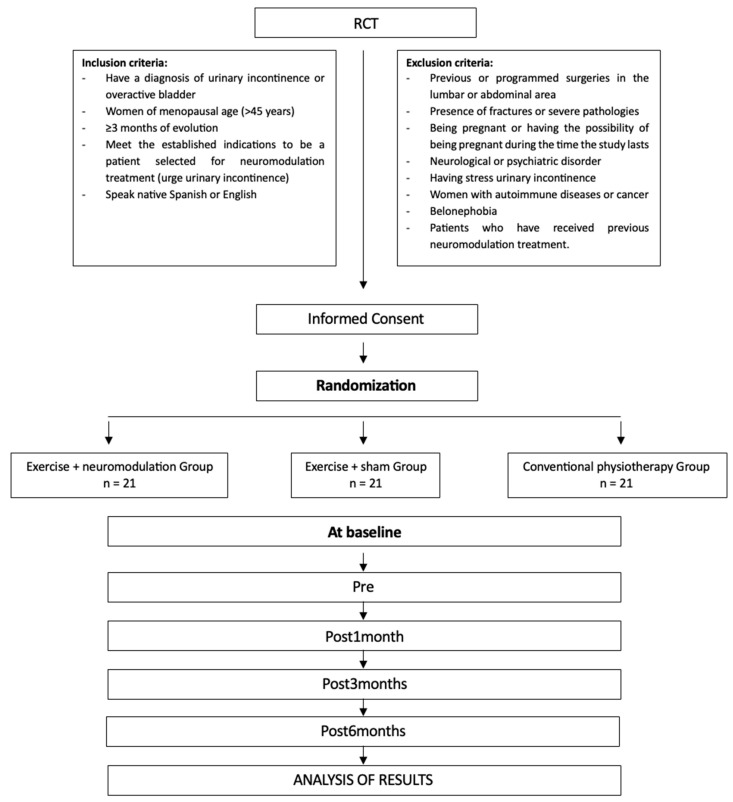
Randomization flow chart and protocol for the intervention measurements.

Once the groups have been assigned, four sample collections will take place at the following time points: Pre (baseline), Post_1month_, Post_3months_, Post_6months_ (Figure 2). All the participants will engage in 24 experimental sessions, each with a duration of approximately 60 min. At each assessment point, the studied variables will be monitored and recorded in the “Data Collection Notebook”. It has been created to systematically record both the primary and secondary outcome variables at each measurement point, in alignment with the study protocol.

## 3. Randomization and Blinding

The principal investigator, supported by a team of experienced physiotherapists with over a decade of expertise in neuromodulation and exercise therapy for UI, will oversee the implementation of the treatment protocol. Stratified randomization will be employed to ensure that the groups being compared are balanced in terms the specific levels of OAB-q SF [25]. The baseline OAB-q SF levels will be assessed before randomization, and the participants will be divided into two strata according to these levels, as outlined in the corresponding sections. One of the researchers will administer the interventions across all three groups, while the other will conduct the outcome assessments to maintain evaluator blinding. A block randomization design (block sizes of 4, 6, or 8) will be applied to ensure an equal number of participants in each group. To minimize the bias, the group allocation will be determined using a randomization table generated by an independent researcher using Excel. A double-blind design will be implemented to ensure objectivity.

## 4. Sample Size

Statistical analysis was performed using a repeated measures analysis of variance (ANOVA), focusing on the primary variable, OAB-q SF. Drawing on a previous study on transcutaneous tibial nerve stimulation in women with idiopathic overactive bladder [26], the Cohen’s effect size was estimated to be 1.04. The statistical power was set at 0.80, with an alpha level of 0.05, considering the three intervention groups. Initially, 54 individuals were allocated to three groups (18 participants per group). To account for a potential 15% dropout rate during follow-up, three additional participants were added to each group, resulting in 21 participants per group and a total sample size of 63. The required sample size was calculated using G*Power software (version 3.1.9.2; Franz Faul, Universität Kiel, Kiel, Germany).

### 4.1. Interventions

#### 4.1.1. Exercise and Neuromodulation Protocol

The experimental group will engage in 90 min sessions, structured into three distinct blocks. In the initial two treatment blocks, the participants perform core stabilization exercises and general strength training, incorporating forced expiration to ensure proper transverse abdominal muscle activation. This approach aims to mitigate the adverse effects associated with increased intra-abdominal pressure. As outlined by Capel-Alcaraz et al. [27], multi-joint exercises for both the upper and lower limbs will be performed using elastic bands or free weights, involving trunk stabilization with abdominal activation during the first 30 min of the session. The session will be initiated with a 10 min mobility and activation block. In accordance with the protocol described by Virtuoso et al. [28], the strength training will adhere to the program established by the American College of Sports Medicine [29]. The exercises will be performed in a rotating sequence, beginning with larger muscle groups to facilitate gradual acclimatization to weight and sessions. As outlined by Helms et al. [30], the rate of perceived exertion (RPE) will be employed as a method to monitor the perceptual responses to training and assess effort during workouts. Zourdos et al. [31] suggest an RPE of 7–10, with a repetition in reserve (RIR) of 3 for resistance strength exercises and subsequent metabolic adaptations. Each set will consist of 10–12 repetitions, with the objective of attaining an RPE of 7–10 based on individual capabilities. Regardless, the number of repetitions will be approximate, with the primary emphasis being on achieving the specified RPE to ensure the intended muscular stimulus (Table 1).

Section 3 focuses on the regulation of anticipatory postural adjustments through the Proprioceptive Postural Reeducation Perineal Method (5P LOGSURF). This method aims to enhance the striated PFM by utilizing reflexive responses associated with sensorimotor control, body awareness, and balance. The exercises will be performed on an unstable oak wood platform that promotes anticipatory muscle activation and continuous postural adjustments. Participants will engage in 30 min sessions divided into two phases, as outlined by Fuentes-Aparicio et al. [15].

##### Neuromodulation Protocol

**Figure 3:**
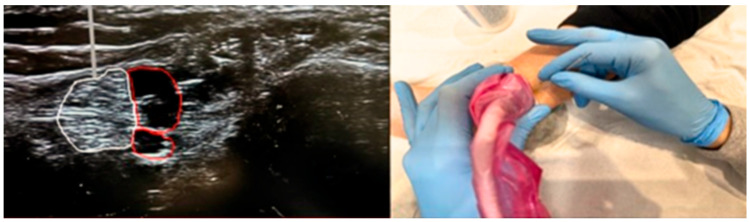
Ultrasound image demonstrating the anatomical localization of the posterior tibial nerve at the level of the medial ankle. The figure includes the appropriate placement and orientation of the ultrasound probe, as well as the in-plane approach typically used for optimal visualization of the nerve (white) and surrounding structures. Additionally, the hypoechoic network observed in the image corresponds to the vascular bundle (red), which accompanies the nerve and includes the posterior tibial artery and vein.

A protocol for electrostimulation of the posterior tibial nerve will be applied to trigger retrograde stimulation through the pelvic nerves linked to the spinal cord via the sacral plexus (S2–S3). The motor branches of the sacral plexus, which are parasympathetic fibers, end in ganglionic cells in the bladder wall. The procedure involves inserting a 34–36 gauge needle at a 60-degree angle to a depth of 3–4 cm, aiming for the posterior tibial nerve located approximately 5 cm (or three finger-widths) above the medial malleolus, just behind the tibia [32,33]. A dispersive adhesive electrode will be positioned near the plantar arch to serve as a ground, connected to the electrostimulator [32,34]. The protocol typically includes 10 sessions (ranging from 6 to 16), each lasting 30 min, conducted 2 to 3 times per week on non-consecutive days over a period of 3 months [34]. The current levels range from 0.5 to 9.0 mA at 20 Hz, with a pulse width of 200–500 ms, adjusted according to the patient’s tolerance, as indicated by discomfort, a tingling sensation in the bottom of the foot, or the bending of the big toe [20]. The patient will either sit or lie in the lateral decubitus position, with the treated leg supported on the table. Anatomical landmarks are employed to ensure precise needle placement 5 cm above the medial malleolus along the path of the posterior tibial nerve. Ultrasound guidance is used to accurately locate the nerve, providing real-time visualization of the anatomical structures and the needle path, thereby ensuring safe and minimally invasive access to deep peripheral nerves [35] (Figure 3). This method, often employed for peripheral nerve blocks in pain management, facilitates effective and targeted stimulations.

#### 4.1.2. Exercise + Sham Neuromodulation Protocol

**Figure 4:**
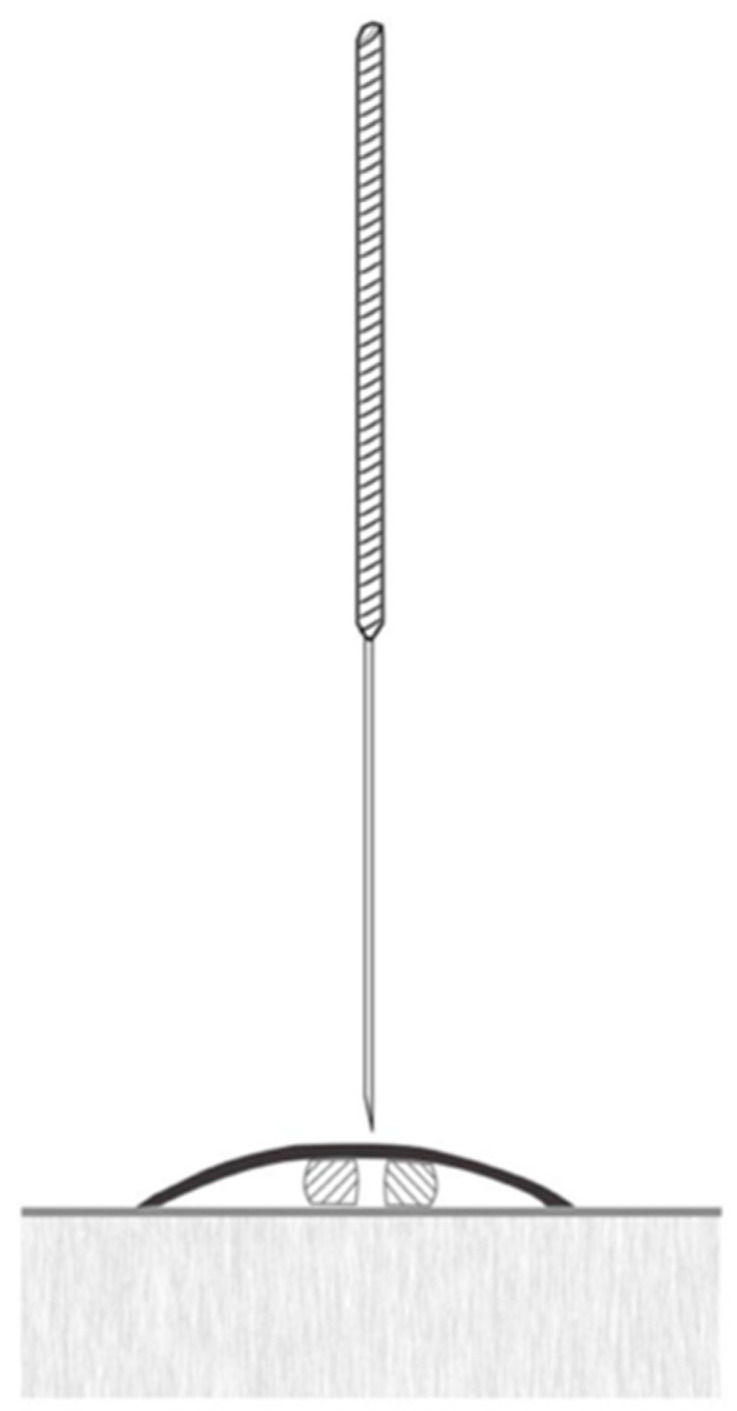
Image of a placebo needle to be used in the study.

In the sham group, a needle with a retractable handle, often utilized in experimental research alongside Streitberger and Kleinhenz placebo needles, will be used. These needles mimic insertion without piercing the skin and can remain in place for the duration of the intervention [36]. The design features a retractable mechanism that pulls the needle back into the handle when pressure is applied, creating the appearance of insertion (Figure 4). This arrangement allows the placebo needle to be swapped with a standard needle, preventing patients from visually distinguishing them. To ensure complete blinding of the treatment, patients will be placed in a lateral decubitus position to block their view of the procedure, preventing them from noticing the differences between the sham and intervention groups. Additional steps will include the use of antiseptic solutions and discarding the needles in visible waste containers to enhance the credibility of the procedure. These measures are consistent with the fundamental placebo principles for masking techniques, as described by Braithwaite et al., 2019 [36].

#### 4.1.3. Conventional Physiotherapy

The control group will undergo a conventional pelvic floor therapy program, primarily focusing on Kegel exercises. This structured regimen includes 8–12 individualized sessions, each lasting between 20 and 40 min and conducted twice per week. The therapy emphasizes precise pelvic floor muscle contractions, ensuring that participants activate these muscles independently without engaging the surrounding areas, such as the abdomen, thighs, or glutes [37]. Unlike the intervention group, which engages in more complex full-body exercises aimed at eliciting higher-level corticospinal responses, this approach excludes strength training and broad movement patterns. Additionally, manual therapy techniques will be employed to relieve trigger points in the pelvic muscles and ligamentous structures, incorporating intracavitary methods to address the mechanical pain.

## 5. Patient-Reported Outcome Measures

### 5.1. Anthropometric Variables

Various assessments and studies concerning body sizes and ratios will be carried out. Information will be gathered on factors such as age, sex, weight, height, job, and the date when the initial diagnosis was made. Furthermore, the overall health condition will be documented to detect any possible comorbidities, along with the educational background of each individual. Patients will also need to disclose whether they are using hormone replacement therapy [26].

### 5.2. Primary Outcome

#### The Overactive Bladder Questionnaire-Short Form (OAB-q SF)

The OAB-q SF is a concise, self-administered instrument featuring two scales that evaluate symptom distress and health-related quality of life (HRQoL) in individuals with overactive bladder [38,39]. This condition-specific questionnaire assesses symptom distress using 6 items and health-related quality of life (HRQoL) through 13 items. Patients rate each item on a 6-point Likert scale. The subscale scores are then combined and converted into a scale from 0 to 100. The symptom distress and HRQoL scales are inversely related: a higher symptom distress score reflects more severe symptoms, while a higher HRQoL score signifies better quality of life [25].

### 5.3. Secondary Outcomes

#### 5.3.1. Mapping of Incontinence Quality of Life (I-QOL)

The I-QOL is a prevalent self-assessment tool for evaluating health-related quality of life in people with urinary incontinence (UI). It includes 22 questions, each scored on a 5-point scale, where 1 = extremely, 2 = quite a bit, 3 = moderately, 4 = a little, and 5 = not at all [40]. These questions are divided into three categories. The sections on Avoidance and Limiting Behaviors consist of 8 questions, Psychosocial Impacts include 9 questions, and Social Embarrassment comprises 5 questions. To calculate the overall I-QOL and subscale scores, the raw item scores are summed and then converted into a 100-point scale, where 0 represents the most severe issues and 100 indicates no problems [41]. This tool has been widely utilized and effectively validated in individuals with UI [42].

#### 5.3.2. Bladder Diary

The bladder diary is a non-invasive assessment tool that provides valuable information about bladder function. It is commonly used to evaluate the frequency and severity of symptoms associated with voiding disorders, such as overactive bladder (OAB) [43]. Additionally, it serves as a method for modifying and improving bladder health habits. Participants complete the diary over a period of 3 to 4 days per week, documenting events continuously for 24 h each day, from morning to night. The records include details of any voiding episodes, the daily fluid intake, and the activities being performed at the time [44].

#### 5.3.3. International Consultation of Incontinence Questionnaire—Urinary Incontinence Short Form (ICIQ-UI-SF)

The ICIQ-UI-SF includes three components that assess subjective frequency, subjective severity, and quality of life through a self-administered questionnaire. Questions 3 to 5 are scored items, with the responses totaled to achieve a minimum score of 0 and maximum of 21 [45].

#### 5.3.4. Electromyography (EMG)

EMG serves as a significant option for monitoring resting muscle tone, muscle strength, and endurance, as well as assessing both typical and atypical functions of the PFM. It is recognized as a reliable and objective technique for muscle evaluation that ensures patient safety [46]. EMG measures the electrical activity generated by the depolarization of muscle fibers and captures changes in the voltage over time. Measurements will be taken multiple times in both the supine and standing positions. Assessments will be carried out with the legs bent and slightly apart. The vaginal probe will be positioned so that the cord points directly forward. Participants will be instructed verbally to contract their PFM with maximum effort, using the following cue: “as if trying to prevent the release of gas.” Each evaluation session will include three sets of contractions, alternating between a 10 s contraction period and a 10 s rest period. Visible muscle activity in the hip adductors, rectus abdominis, or gluteal areas will not be allowed [47].

#### 5.3.5. Oxford Scale

The Oxford Scale is a subjective classification system used to assess the strength and quality of PFM contractions through intracavitary palpation with one or two fingers. The modified scale includes 5 grades: 0 = no contraction; 1 = flickering muscle movements; 2 = weak contraction; 3 = increased pressure with slight muscle elevation; 4 = firm contraction with moderate elevation of the vaginal posterior wall; and 5 = strong contraction with finger resistance against the abdominal wall [48].

#### 5.3.6. Tampa Scale 11 (TSK-11)

Kinesiophobia is characterized as “an excessive, irrational, and debilitating fear of physical movement and activity stemming from a sense of vulnerability to a painful injury or re-injury,” and it is particularly relevant to individuals with chronic pain who fear movement [49,50]. The TSK is a self-report scale designed to measure fear related to movement. Initially, the scale had 17 items, but the most commonly used version is now the 11-item TSK-11 [51]. Each item is scored on a 4-point Likert scale, ranging from 1 (“strongly disagree”) to 4 (“strongly agree”). The total score ranges from 11 to 44, with higher scores indicating a greater fear of movement-related injuries [52]. Assessing kinesiophobia is crucial because physical exercise plays a vital role in rehabilitation, and high levels of kinesiophobia can hinder treatment adherence [53]. Furthermore, the scale has demonstrated strong psychometric properties, with a reliability coefficient of 0.84 and a 90% confidence interval [52].

#### 5.3.7. Pain Catastrophizing Scale (PCS)

In this study, the Spanish adaptation of the Pain Catastrophizing Scale (PCS) will be utilized to evaluate pain catastrophizing. The PCS is a self-report instrument designed to measure catastrophic thoughts related to pain. It consists of 13 items that are categorized into three domains: rumination, magnification, and helplessness. Each item is rated on a scale from 0 (not at all) to 4 (all the time), with a total possible score ranging from 0 to 52 [54]. This version has demonstrated strong psychometric properties, including a Cronbach’s alpha (α) of 0.95 and test–retest reliability coefficients of 0.75 over six weeks and 0.70 over ten weeks [55]. The validated Spanish version will be used in this research.

## 6. Program Feasibility and Safety: Attendance and Compliance

Adherence to exercise regimens for UI presents a multifaceted challenge, necessitating both behavioral modification and active patient engagement [56]. The process of behavioral change, essential for effective treatment adherence, is often intricate [57]. Empirical evidence indicates that home-based interventions are less efficacious compared to those supervised by a physiotherapist with specialized training [58]. Specifically, a structured exercise program for UI, when supervised during the treatment phase, is associated with higher adherence rates in both the short and long term [59]. Moreover, non-adherence to treatment poses a significant barrier to the success of UI interventions, with the efficacy potentially diminishing substantially if compliance falls below 80% [60]. Consequently, adherence is a critical determinant of the treatment efficacy and the enhancement of quality of life [61]. This study will implement an exercise regimen aimed at enhancing core stabilization and overall strength by incorporating forced exhalation to activate the transverse abdominal muscle, thereby mitigating the adverse effects of increased intra-abdominal pressure. In both the intervention and placebo groups, the training program will be conducted under direct supervision following posterior tibial nerve neuromodulation. Participants in the exercise groups will adhere to the prescribed training frequency, as monitored by the SIRAS scale [59].

## 7. Oversight and Monitoring

This study was collaboratively designed by researchers J.V.M. and J.L.G., with the participation of a third researcher, P.B.G., who will also contribute to its execution. Together, they will be responsible for coordinating patient participation, administering treatments, and monitoring adverse events or protocol deviations. Both the participants and the therapists will be instructed to promptly report any adverse events, discomfort, or deviations from the protocol. All incidents will be documented and reviewed by the research team. In the case of any serious adverse events, the principal investigators will immediately inform the Research Ethics Committee of the Catholic University of Valencia. Continuous patient monitoring will be implemented throughout the study to ensure the timely identification and management of potential risks.

## 8. Data Collection and Analysis

### 8.1. Data Collection

The data collection plan has been meticulously designed to guarantee the quality, integrity, and confidentiality of the data collected throughout the randomized clinical trial. The study coordinator will oversee data management and assume responsibility for specific tasks following the collection of paper-based records. Subsequently, encrypted Excel databases will be created to maintain participant confidentiality by implementing a double-entry procedure to ensure the integrity and accuracy of the records. Key methodological aspects, including the sample size, eligibility criteria, randomization, blinding procedures, treatment, and outcome variables, will be precisely defined and detailed in the methodology section of the study. Data will be collected using standardized record forms and electronic systems, with a rigorous protocol for eliminating incomplete entries, to ensure data consistency and reliability at the conclusion of the trial.

### 8.2. Statistical Analysis

#### 8.2.1. Baseline Characteristics

For the comparison of the results between groups, inferential analysis will be conducted using a repeated measures ANOVA or chi-square tests to determine whether there is a significant relationship between two categorical variables.

#### 8.2.2. Analysis of the Outcome Measures

The assumption of normality will be evaluated using the Kolmogorov–Smirnov test, while the homogeneity of variances will be assessed with Levene’s test. A two-way mixed-design ANOVA will be employed, with the time serving as the within-subject factor and the treatment group as the between-subject factor. Tukey’s post hoc correction will be applied to examine the effects of both the primary and secondary outcomes. The results will be presented as the mean differences (MDs) with 95% confidence intervals (CIs). The effect sizes (ESs) will be calculated using Cohen’s d. In the event of participant dropouts during the study, or if the statistical power falls below the predetermined threshold of 80%, an intention-to-treat analysis will be conducted [62]. All the statistical analyses will be conducted by an independent researcher who will not be involved in the data collection and will utilize anonymized, coded datasets.

## 9. Dissemination Plan

The primary objective of a dissemination plan is to ensure that research findings and outcomes are effectively communicated, understood, and applied, thereby maximizing their potential impact on the field. A well-structured dissemination plan is crucial to engaging various stakeholders and ensuring that research is accessible and actionable within the relevant field. In this context, the overarching goals of the dissemination plan include the widespread dissemination of research results, ensuring that these findings are made available and comprehensible to an informed audience with expertise in the study area. This can be accomplished through multiple channels, such as academic publications, specialized workshops, and accessible patient or public information. Furthermore, a key aim of the dissemination plan is to actively promote the practical application of the research findings. This involves influencing relevant stakeholders, including policymakers, practitioners, and the scientific community, to integrate the findings into their work. In doing so, the plan seeks to enhance the understanding of the research, guide future research directions and impact policy and practice on a broader scale. Through these efforts, this plan endeavors to ensure that the research contributes meaningfully to both the academic field and real-world applications.

## 10. Discussion

Treatments for OAB and UUI have been evaluated in multiple studies, leading to a transition toward combined approaches, as individual interventions demonstrate limitations in terms of the efficacy and long-term adherence [6,8]. In this context, the combination of peripheral neuromodulation, particularly through PTNS, with PFMT is proposed as a potentially more efficacious strategy to ameliorate the symptoms and enhance the quality of life in patients [10,17]. The evidence analyzed indicates that neuromodulation acts on the sacral circuits (S2–S3), modulating involuntary detrusor muscle contractions and reducing urinary urgency, frequency, and incontinence episodes, with response rates ranging from 55% to 71% [33,63]. Conversely, PFMT, which encompasses supervised exercises that increase awareness and strengthen the pelvic floor muscles, contributes to improved muscle strength and neuromuscular control, facilitating urethral closure and inhibitory reflex responses during episodes of urgency [64]. However, there is an emphasis on the necessity of implementing multidimensional strength training programs that integrate both voluntary muscle contraction and functional and involuntary movements [13,65], in addition to complementary techniques such as anticipatory postural control and proprioceptive re-education (5P^®^ LOGSURF) [15]. In this regard, the present protocol will be developed as a 12-week randomized controlled trial, aiming to elucidate the efficacy of combined interventions and establish standardized protocols to ensure rigorous follow-up of clinical outcomes [17,22]. These time points (1, 3, and 6 months) were chosen to allow for the evaluation of both the short- and medium-term effects of the intervention in the present study. Lastly, numerous studies have shown that a score variation ranging from 2.1% to 16.5% can be deemed clinically significant, depending on the specific tool employed and the context of the intervention [66].

## Figures and Tables

**Table 1:** The table shows the standard planning for each session proposed for the following study.

	Exercise	Sets	Repetitions	Intensity	Rest	Effort
WU (Warm-Up)	Cat–Camel	1	10–12	-	-	-
	Quadruped Trunk Rotation	1	10–12	-	-	-
	Mahoma	1	10–12	-	-	-
B1	Crunch	3	10–12	7–10 RPE	60″	Moderate
	Goblet squat	3	10–12	7–10 RPE	60″	Moderate
	ISO Lunge + Row	3	10–12	7–10 RPE	60″	Moderate
B2	Dead bug	3	10–12	7–10 RPE	60″	Moderate
	Deadlift	3	10–12	7–10 RPE	60″	Moderate
	Box Step-Up + Shoulder Press	3	10–12	7–10 RPE	60″	Moderate

## Data Availability

The data presented in this study will be available on request from the corresponding author.

## Abbreviations

The following abbreviations are used in this manuscript:

ICS	International Continence Society
OAB	Overactive bladder
UUI	Urge urinary incontinence
DO	Detrusor overactivity
PFM	Pelvic floor muscle
PTNS	Percutaneous tibial nerve stimulation
PFMT	Pelvic floor muscle training
PTNN	Posterior tibial nerve neuromodulation
RCT	Randomized controlled trial
RPE	Rate of perceived exertion
RIR	Repetition in reserve
WHO	World Health Organization
